# Supplementary material for: Ultra-high electrostriction and ferroelectricity in poly (vinylidene fluoride) by ‘printing of charge’ throughout the film
Source: Nat Commun. 2025 Jan 16;16:744. doi: 10.1038/s41467-025-56064-w (PMC11739654; doi:10.1038/s41467-025-56064-w)
Supplement: Supplementary file 1 — Supplementary Information [file 41467_2025_56064_MOESM1_ESM.pdf]

# Supplementary Information

# Ultra-high electrostriction and ferroelectricity in poly (vinylidene fluoride) by 'printing of charge' throughout the film

Ningyi Zhang[1,2]*, Xiaobing Dong[1,2], Shihui He[1,2], Zhao Liang[1,3], Weipeng Li[1,2], Qihao Qian[1,2], Chao Jiang[1,2]*

[1]*State Key Laboratory of Advanced Design and Manufacturing Technology for Vehicle, College of Mechanical and Vehicle Engineering, Hunan University, Changsha, 410082, China.*

[2]*Key Laboratory of Advanced Design and Simulation Technology for Special Equipment, Ministry of Education, College of Mechanical and Vehicle Engineering, Hunan University, Changsha, 410082, China.*

[3]*Institute of Micro/Nano Materials and Devices, Ningbo University of Technology, Ningbo, 315211, China.*

*Corresponding author. Email: nzhang@hnu.edu.cn (Ningyi Zhang); jiangc@hnu.edu.cn (Chao Jiang)

**Table of Content**

**Note 1.** Printing method and parameters

**Note 2.** Sample numbers and corresponding parameters

**Note 3.** The use of mixed powders

**Note 4.** Verification of the ultra-high electrostrictive strain in BUE-PVDF films

**Note 5.** The conductivity and charge leakage issue of the printed PVDF

**Note 6.** Ruling out the contribution of the Maxwell stress

**Note 7.** The *D-E* curve of the PVDF under varied electric field

**Note 8.** The variation on different methods for the measurement on permittivity

**Note 9.** Changes in porosity and dielectric property with ink concentration

**Note 10.** The storing of the printed charges on pore surfaces

**Note 11.** The pore structure and surface potential for high-permittivity samples

**Note 12.** Evaluating the effect of pore size on the dielectric property of BUE-PVDF

**Note 13.** The influence of crystallinity on the surface potential and dielectric property

**Note 14.** The comparison with casted PVDF and commercial PVDF

**Note 15.** Estimation of ferroelectric and electrostrictive performances in BUE-PVDF from *S-E* curves

**Note 16.** Previous methods for the improvement on the electrostrictive property in PVDF

**Note 17.** The linear relation between permittivity and optimized electrostriction in BUE-PVDF

**Note 18.** The characteristic of pore structure in the high-permittivity PVDF samples

**Supplementary Figures** S1 to S14

**Supplementary Tables** S1 to S6

**References**

**Supplementary Note 1. Sample numbers and corresponding parameters.**

The sample 1 series, as shown in Supplementary Table S1, corresponds to Fig. 1B and reflects the change of the crystal phase fraction in the printed PVDF films under different voltages. Other printing parameters include: PVDF powder with $M_w$ of 275000 g mol$^{-1}$; ink with PVDF concentration of 14 wt.% in NMP; substrate temperature of 60 °C; relative humidity of 25%; printing time of 1 h.

Samples 2 series, as shown in Supplementary Table S2, are solvent-casted (painting the PVDF ink on the substrate and curing it to produce the PVDF film) and printed respectively to obtain TSD test results (corresponding to Fig. 2B). The same ink is used for two samples: PVDF powder with $M_w$ of 275000 g mol$^{-1}$; ink with PVDF concentration of 14 wt.% in NMP. The same curing temperature of 60 °C are used for both samples. These PVDF films are heated linearly in an electronically controlled oven at a speed of 3 °C min$^{-1}$. A circular induction electrode with a diameter of 50 mm is mounted 3 mm above one of the non-metalized surfaces of the charged film. The discharge current between this electrode and the sample holder is determined by a sensitive electrometer (Keithley 428). The actual temperature is measured with a PT1000 temperature sensor. The temperature and discharge current are recorded to derive the TSD curve by a data acquisition recorder, Keithley-6517B. This result provides information about the charge released from different depths in the film [1].

The samples 3 and 4 series, as shown in Supplementary Table S3, correspond to PVDF films with different thicknesses by varying the printing time in Fig. 2C and Fig. 2D, respectively. Different ink concentrations can lead to different charge densities in series 3 and 4. Other printing parameters are the same: PVDF powder with $M_w$ of 275000 g mol$^{-1}$; substrate temperature of 40 °C; relative humidity of 22%; printing voltage of 4 kV. Then, these samples' surface potential and permittivity are measured by KPFM and impedance method, respectively.

The sample 5 series, as shown in Supplementary Table S4, corresponds to PVDF samples in Fig. 2E with different concentrations from 10 wt.%-14 wt.% in NMP. We adjust the printing time to obtain the films with the same thickness. Other printing parameters are also the same, including the PVDF powder by 1:1 mixing of two PVDF powder with $M_w$ of 275000 and 534000 g mol$^{-1}$ respectively; substrate temperature of 70 °C; relative humidity of 30%; printing voltage of 6 kV. It shows that the dielectric property of the material can be well controlled by varying the

concentration of the ink. In this study, we mainly use the ferroelectric analysis method to characterize the relative permittivity instead of the commonly used impedance analysis because this method can better reflect the dielectric properties of the material, including at both lower and higher electric fields.

In the sample 6 series, as shown in Supplementary Table S5, corresponding to Fig. 2H, we adjust multiple parameters to obtain the PVDF films with different relative permittivity to exhibit the influence of dielectric property on the electrostrictive coefficient $M_{33}$ of materials. Two printing parameters are the same: PVDF powder with $M_w$ of 275000 g mol$^{-1}$ and a printing voltage of 4 kV. The relative permittivity and strain of the material are obtained from ferroelectric analysis. For ferroelectric analysis of PVDF films, we first sputter gold electrodes on both sides of the surface to form a simple sandwich sensor. Then, AC voltage is applied to both sides of the sensor. A photonic sensor detects the thin-film strain, and the hysteresis loop of the printed sample is measured by the Sawyer-Tower circuit.

**Supplementary Note 2. Printing method and parameters.**

The EHD printing platform in this study is composed of an FDM printer with X and Y tables to control the position of the needle on the printing plate. As shown in Supplementary Fig. S1, the needle is connected to a high-voltage power supply with DC voltages ranging from 3 kV to 8 kV. This EHD printing system operates with an accuracy of 50 μm, and the nozzle-substrate distance is about 1-2 mm. The substrate must be heated during printing to ensure the current slice is solidified (or semi-solidified) before printing the next slice. The substrate temperature is set to 40-80 °C. Besides, adding acetone (0-10 wt.%) can accelerate the volatilization process and affect curing. Manipulating these two parameters allows the control of curing time and thus guarantees continuous printing.

Generally, two types of PVDF powder are selected with $M_w$ of 534000 g mol$^{-1}$ (Kynar® 761 powder) and 275000 g mol$^{-1}$ (Kynar® 721 powder), respectively. N-methyl-2-pyrrolidone (NMP) and N,N-Dimethylformamide (DMF) are used as polar organic solvents to prepare PVDF solutions with different concentrations ranges of 8-12 wt.% and 12-16 wt.% for Kynar® 761 powder and Kynar® 721 powder, respectively. Other processing parameters include needle size (23#-30#), and humidity (20%-70%).

**Supplementary Note 3. The use of mixed powders.**

PVDF powder with 275,000 g $mol^{-1}$ molecular weight (Kynar® 721) is widely used as raw material due to its wide range of sources, low price, and stable performance[2, 3]. However, the dielectric property of film entirely made by Kynar® 721 powder is slowly increased with the adjustment of ink concentration (Supplementary Fig. S2). Therefore, we attempt to mix Kynar® 721 powder with a higher molecular weight of 534,000 g $mol^{-1}$ (Kynar® 761) to better adjust the internal pore structure (Supplementary Fig. S2). Consequently, we can successfully present a strong effect of ink concentration on the dielectric property of BUE-PVDF in Fig. 2E.

In the mixing process, Kynar® 721 powder and Kynar® 761 powder with the same weight is well mixed. Then, the corresponding solvent is added to the mixed powder to obtain specific concentrations of PVDF ink solutions with a mixing weight ratio of 1:1.

Supplementary Fig. S2A presents the dielectric property of the samples made by single powder ($M_w$=275,000 g $mol^{-1}$) in their printable concentration range. Note that this series of samples is identical to those shown in Supplementary Fig. S4. The relative permittivity values of the single powder BUE-PVDF are ranged between 12.5-17.5, while that values from their mixed powder counterparts are ranged between 18-36. These limited permittivity values in single powder BUE-PVDF could be explained by their pore structures in Supplementary Fig. S2D-S2E. Due to their similar size in PVDF particles, the pore sizes of single powder samples are much lower than their mixed powder counterparts as shown in Supplementary Fig. S7. Therefore, the much lower density of small and medium pores in single powder samples also leads to a lower permittivity. Meanwhile, since the single powder PVDF becomes sticker and less fluent with an increased concentration, the pore size of the 12 wt.% sample (Supplementary Fig. S2B and S2C) is higher than that of the 15 wt.% sample (Supplementary Fig. S2D and S2E). Also, the shortage of ink material at the gap area (Supplementary Fig. S2E) in a high-concentration sample may tend to form large pores and even macroscopic pores (Supplementary Fig. S4E).

In contrast, the PVDF ink with mixed powder tends to produce small pores during solidification due to the significant difference in size between the two PVDF powders. Therefore, the mixed sample can achieve a higher and broader range of permittivity value with many small and medium pores adjusted by the ink concentrations (Supplementary Fig. S2). Consequently, the ink with mixed powder is adopted in Fig. 2E to demonstrate better control through single printing parameter, i.e., PVDF concentration on the dielectric property of BUE-PVDF.

**Supplementary Note 4. Verification of the ultra-high electrostrictive strain in BUE-PVDF films.**

In this study, we mainly use the optical method by photonic sensors to measure the induced strain of PVDF films under the electric field. This method is based on the reflection of monochromatic light[4] and is already applied in electroactive polymers[5]. Meanwhile, the photonic sensors by MTI Instruments Inc. (Albany, NY) are used in this method. In principle, it can measure displacement statically from 10 nm to 5 mm or at frequencies up to 150 kHz[4]. However, the electrostrictive displacement of widely used PVDF films under a low field (<10 MV $m^{-1}$) is usually lower than 10 nm. Thus, another method should also be introduced to guarantee the accurate measurement of the tiny strain.

Here, we construct a piezoresponse force microscopy (PFM) test system (Sinometer, model: HY3020E AFM) based on the AFM platform, which has been successfully applied to probe the electro-mechanical (EM) properties of inorganic ceramics and biological materials[6]. The basic idea of PFM is to introduce a localized electric field on the surface of the piezoelectric sample and evaluate the corresponding deformation through the measurement of displacements. As shown in Supplementary Fig. S3A, the amplified harmonic signal is applied on the electrode of the PVDF film to induce the strain along the film's thickness direction. To quantify the induced displacement, we employ a dual ac resonance tracking piezoresponse force microscopy (DART-PFM), which permits us to probe the displacement with a spatial resolution up to sub-micrometers[7]. The diameter of the PFM cantilever tip used to perform piezoelectric experiments is a silicon probe coated by conductive Platinum-Iridium ($P_tI_r$) with a diameter of 50 nm. The system in this study has been operating with an AC bias voltage of 150 V and a frequency of 0.1 Hz. The vertical pressure response ($d_{33}$ mode) is measured only in the vertical scan mode. In addition, we hold the tip in a fixed position to detect its real-time displacement along the thickness direction instead of the scanning pattern used in a typical PFM test.

PFM tip calibration and spring constant measurements are performed before formal experiments to transform the raw data of voltages into displacement value. Here, we select piezoelectric ceramic PZT as the standard sample with a $d_{33}$ value of 500 pm $V^{-1}$. The voltage with a 1-10 V, 0.1 Hz harmonic AC signal is applied along the thickness direction of the PZT

sample. By monitoring the displacement of the PZT sample (converted from the input voltage and its $d_{33}$ value) with the output signal from the AFM platform in Supplementary Fig. S3B, we can determine the linear relationship between this output signal and the corresponding displacement in this PFM system with a slope of 0.167 V nm$^{-1}$.

Later, the applicability of the PFM method in electroactive polymers is examined by a commercial LDT1-028K PVDF sample (MEAS) (with a thickness of 28 μm). As shown in Supplementary Fig. S3C, the resolution of measured strains for the polymers by the PFM method can be as low as 0.0003% (equivalent to 0.09 nm). Meanwhile, the measured $d_{33}$ of this commercial PVDF through the PFM method is 17.8 pm V$^{-1}$, a little lower than its nominal value of 22 pm V$^{-1}$. Therefore, it is feasible to compare the strain differences between traditional PVDF and BUE-PVDF by the PFM method under a low electric field.

The BUE-PVDF sample exhibits much higher field-induced strain (Supplementary Fig. S3D) than commercial piezoelectric PVDF under a low electric field. At a field strength of 1.7 MV m$^{-1}$, the strain of BUE-PVDF can reach 0.02%, seven times that of commercial PVDF of 0.003%. In addition, the $M_{33}$ value measured by the PFM method is 87.1×10$^{-18}$ m$^{2}$ V$^{-2}$, similar to the value obtained by the photonic sensing method for the same sample (98.6×10$^{-18}$ m$^{2}$ V$^{-2}$). Therefore, the results of photonic sensing in the manuscript can satisfactorily agree with that of PFM. These two methods collectively confirm that BUE-PVDF film has a much higher electrostrictive strain (Supplementary Fig. S3) than ordinary PVDF.

**Supplementary Note 5. The conductivity and charge leakage issue of the printed PVDF.**

There is a concern that increasing the number of large pores can significantly enhance the conductivity of PVDF. Jin *et al*. pointed out that a widened *D-E* curve with the same slope can be used to detect the increased conductivity and the risk of charge leakage[8]. To verify the effect of pore-induced conductivity on dielectric property, we designed a batch of PVDF film samples (made by Kynar® 721 powder) with different porosities. As shown in Supplementary Fig. S4A, the corresponding residual electric displacement of the three samples with 12 wt.%, 14 wt.%, and 16 wt.% PVDF concentration are 0.0017 μC cm$^{-2}$, 0.0029 μC cm$^{-2}$, and 0.1476 μC cm$^{-2}$, respectively. Therefore, the *D-E* curve gradually widened with the increasing PVDF concentration. Since the applied electric field is always less than 6 MV m$^{-1}$, it is much lower than

the threshold electric field required to activate the $\beta$ phase[9]. Therefore, the widening phenomenon in Supplementary Fig. S4A should be due to the enhancement of the overall conductivity.

To further investigate this widening phenomenon, we compare the pore structure of the samples with distinct residual electric displacement. For the samples with less residual electric displacement (12-14 wt.% PVDF concentration) in Supplementary Fig. S4A, we cannot find any large pores (Supplementary Fig. S4B), and the size of the existing pores is usually less than 2 μm (Supplementary Fig. S4C). In contrast, for the sample with the most widened *D-E* curve (16 wt.% PVDF concentration) in Supplementary Fig. S4A, there are many macroscopic pores with a size larger than 50 μm widely distributed in the gap area (Supplementary Fig. S4D and S4E). This significant difference in pore structure between Supplementary Fig. S4D and Fig. S4E is highly likely due to the less fluent of the printed droplet at a higher PVDF concentration. During solidification, the droplet with higher concentration becomes more reluctant to flow from the track area to the gap area. Thus, the shortage of material at the gap area may tend to form macroscopic pores or even through holes, resulting in an apparent widening of the *D-E* curves.

Nevertheless, the relative permittivity of all samples in Supplementary Fig. S4A is around 14 (the slope of the gray region) despite their significant differences in conductivity. Their similarity in permittivity proves that the change in the overall conductivity does not influence the dielectric property of BUE-PVDF. In this work, the enhanced permittivity of BUE-PVDF is achieved by the high density of small and medium sized pores (Fig. 2E) with their size smaller than 10 μm (Supplementary Fig. S7). Considering the much smaller pore size and narrower *D-E* curve than that in the 16 wt.% sample in Supplementary Fig. S4A, we believe that the increase of dielectric property in BUE-PVDF should not stem from the pore-induced extra conductivity.

As Li *et al.* noted[10], the electret charged through a macroscopic porous structure will bring the risk of charge loss. This phenomenon has also been found in Supplementary Fig. S4: the risk of severe leakage, i.e., the dramatic widening of the hysteresis curve in Supplementary Fig. S4A, is due to macroscopic pores in Supplementary Fig. S4E. Therefore, the charge loss may occur in BUE-PVDF like ordinary electret materials. However, ordinary electrets rely on macroscopic pores to inject charge, so their leakage is unavoidable. In contrast, BUE-PVDF mainly relies on small and medium pores to preserve a high density of charge during the printing process

(Supplementary Fig. S8), thus the pore structure in BUE-PVDF will not necessarily lead to charge loss.

Meanwhile, Supplementary Fig. S4A proves that the dielectric property in BUE-PVDF do not considerably increase with the number of macropores and the extent of leakage. In other words, the improved dielectric property in BUE-PVDF (Fig. 2E) are not due to the macroscopic pore structure (Supplementary Fig. S7). The risk of leakage in BUE-PVDF can be avoided by carefully controlling the pores' size through adjusting printing parameters like ink concentration (Supplementary Fig. S2, Fig. S4).

**Supplementary Note 6. Ruling out the contribution of the Maxwell stress.**

Despite the electrostrictive effect, Maxwell stress also contributes to the strain and is also proportional to the square of the electric field/polarization [11, 12, 13]. The difference between the strains induced by electrostriction and the Maxwell stress is that the former is usually induced by the potential anharmonicity of the ionic crystal, while the latter originates from free charges close to the electrodes. Therefore, we need to evaluate the impact of Maxwell stress on the field-induced strain. As an electric field is applied to the electrodes on the dielectrics, the electrostatic force resulting from the free charges squeezes or stretches the materials. This electrostatic force is the Maxwell stress, which occurs in all insulators under an applied electric field [14]. The longitudinal strain for dielectrics induced by a Maxwell stress is given by [11]:

$$S_M = -\frac{1}{2}s\varepsilon E^2 = -\frac{1}{2}s\varepsilon_r\varepsilon_0 V^2/t^2\ , \qquad \text{(S1)}$$

where $S_M$ is the longitudinal strain induced by the Maxwell stress, $s$ is the elastic compliance of the material, $\varepsilon_r$ is the relative permittivity, $\varepsilon_0$ is the vacuum permittivity constant ($8.85\times10^{-12}$ F $m^{-1}$). The values of $s$, $\varepsilon_r$ are obtained to be $8.7\times10^{-10}$ $Pa^{-1}$, and 8.8 by stress-strain plot and *D*-*E* plot, respectively. Substituting the *V* value of 150 V, and *t* value of 40 μm into Eq. (S1), the maximum absolute value of Maxwell strain in this study can be determined ($4.77\times10^{-7}$). In contrast, the strain value at the same voltage obtained from Supplementary Fig. S3D is about $1.1\times10^{-3}$, more than three orders of magnitude higher than the Maxwell strain. Thus, the quadratic strain obtained under the electric field in BUE-PVDF is induced by the electrostrictive effect rather than the Maxwell stress.

**Supplementary Note 7. The *D-E* curve of the PVDF under varied electric field.**

Supplementary Fig. S5 compares the hysteresis curves of commercial PVDF and BUE-PVDF under different applied cyclic electric fields. Typically, the $\beta$ phase in PVDF can only be activated when the amplitude of the applied electric field exceeds a particular threshold value[9]. For instance, when the electric field amplitude exceeds 88 MV $m^{-1}$ (Supplementary Fig. S5A), the $\beta$ phase of commercial PVDF is activated, and its hysteresis curve exhibits a typical ferroelectric style: the sample is polarized and maintains a high remanent polarization as the electric field becomes zero. In contrast, when the amplitude of the applied electric field is less than 60 MV $m^{-1}$ (Supplementary Fig. S5B), the $\beta$ phase of commercial PVDF cannot be effectively activated. At this time, the electric displacement $D$ of the sample is approximately linearly increased with the applied electric field $E$[8, 15], which is similar to the hysteresis curve of dielectric materials.

In contrast, this work significantly reduces the threshold electric field (coercive field) to activate the $\beta$ phase in the BUE-PVDF sample to less than 40 MV $m^{-1}$ (Supplementary Fig. S5C). This large discrepancy in threshold electric field between commercial PVDF and BUE-PVDF should probably related to the electro-lubricating effect. With the printed charge stored throughout the film, the BUE-PVDF successfully minimized the required applied field to achieve a 7 μC $cm^{-2}$ electric displacement from 112 MV $m^{-1}$ in Supplementary Fig. S5A to 46 MV $m^{-1}$ in Supplementary Fig. S5C. In addition, the BUE-PVDF exhibits much higher permittivity in Supplementary Fig. S5D (29.2 on average) than normal PVDF, which is 2.5 times higher than the average relative permittivity of commercial PVDF in Supplementary Fig. S5B.

**Supplementary Note 8. The variation on different methods for the measurement on permittivity.**

Theoretically, the impedance method measures the dielectric properties in different frequency ranges at low electric fields, while the ferroelectric method measures the average dielectric property through different electric fields at the same frequency[8, 16]. To verify the difference between these two methods in dielectric measurements, we added the measuring results by impedance analysis for all samples in Fig. 2E. As shown in Supplementary Fig. S6A, both methods provide consistent measuring results for the samples with various PVDF concentrations. However, the values from ferroelectric analysis are always higher than their impedance analysis

counterparts. Further comparison among these samples exhibits a proportional relationship between the dielectric measurement results of the two methods in Supplementary Fig. S6B. There could be two reasons for the 40% higher value obtained from the ferroelectric method. Firstly, the measurement frequency of the impedance method is 100 Hz, which is much higher than the 10 Hz measurement frequency for the ferroelectric method. Thus, the increase in frequency results in a lower dielectric value[17]. Secondly, the applied voltage of the impedance method is only 0.5 V, which is much lower than the voltage range of the ferroelectric method (0-100 V). Lower voltages also have an impact on dielectric results[15].

**Supplementary Note 9. Changes in porosity and dielectric property with ink concentration.**

Supplementary Fig. S7 summarizes SEM images of the pore structure in BUE-PVDF samples with varied ink concentrations. As the ink concentration increases from 10 wt.% to 14 wt.%, the variation for relative permittivity exhibits a trend of increasing first and then decreasing. The sample with the lowest ink concentration (10 wt.%) has the lowest relative permittivity (about 18, Supplementary Fig. S7A). Despite many large pores in the gap area (Supplementary Fig. S7B), most regions in this sample exhibit high density without any noticeable pores (Supplementary Fig. S7C). The proportion of this high-density region continues to decrease as the ink concentration (Supplementary Fig. S7D) increases, reaching its minimum with the 12 wt.% ink concentration (Supplementary Fig. 7G). Meanwhile, the relative permittivity $\varepsilon_r$ enhances to 36 in the 12 wt.% concentration sample. With the further increase of ink concentration (Supplementary Fig. S7J), the proportion of high-density regions starts to increase, along with the reduction of permittivity. For the 14 wt.% concentration sample (Supplementary Fig. S7M), the relative permittivity $\varepsilon_r$ is reduced to 18.

SEM images at high magnification compare the change of pore morphology with varied ink concentrations to investigate further the effect of porosity on the dielectric property of BUE-PVDF. When the sample only has large pores (over 10 μm, Supplementary Fig. S7B, and Supplementary Fig. S7N) and few small pores (0.5-3 μm, Supplementary Fig. S7C, and Supplementary Fig. S7O), the relative permittivity $\varepsilon_r$ of the material is 18. This value is much higher than that of the casted sample without any stored charge (2.5, as shown in Fig. 3D). Since a significant number of large pores appear in all samples with $\varepsilon_r$ values above 18 (Supplementary Fig. S7B, E, H, K, and N), the enhanced $\varepsilon_r$ value in Supplementary Fig. S7A may result from the

large pores.

However, the large pores are not sensitive to the change of permittivity in Supplementary Fig. S7. Comparing the magnified images of the high-density region, the material in the track areas is almost wholly fused when the ink concentration is either too low (10 wt.%, Supplementary Fig. S7C) or too high (14 wt.%, Supplementary Fig. S7O). In contrast, many small pores are reserved in the track areas after the BUE-PVDF printing with moderate concentrations (11-13 wt.%, Supplementary Fig. S7F, I, and L). These small pores reduce the proportion of high-density regions and provide many inner surfaces to store the printed charge. Therefore, the further enhanced permittivity ($\varepsilon_r$=36) in the sample with medium concentration should result from the small pores (Supplementary Fig. S7I). In other words, pores with smaller sizes are more effective in improving the dielectric property of BUE-PVDF.

**Supplementary Note 10. The storing of the printed charges on pore surfaces.**

Supplementary Fig. S8 shows the correspondence between the surface morphology and the surface potential of different samples. Since the surface depressions imply the presence of pores, the morphology on the surface of samples can approximately reflect the size and number of pores. Generally, the regions on the surface can be classified into three types according to the localized density, as shown in Supplementary Fig. S8A: the low-density region (L1 blue circle) with a single large pore, the medium-density region (M1 green circle) with multiple small pores, and the high-density region (H1, H2 yellow circle) without any macro-pores.

The comparison between the surface height map (Supplementary Fig. S8A) and the surface potential diagram (Supplementary Fig. S8D) verifies the storing effect of the printed charges on pore surfaces. For instance, the low-density region (L1) has a lower surface potential. However, the higher localized potential near the bottom side of L1 indicates the storage of charges near the pore surface (a distance lower than 1 μm). Also, the increase of surface potential in the medium-density region (M1) is probably due to storing charges by the multiple small pores among the particles. Besides, the high-density region (H1) with sufficiently fused particles and much fewer pore surfaces has a lower surface potential, suggesting the effect of pore surface on storing the printed charges.

The other two samples (Supplementary Fig. S8B and S8E, and S8C and S8F) exhibit similar trends of charge storing on pore surfaces as described above (Supplementary Fig. S8A and S8D).

More importantly, the comparison among the three samples reveals that the absolute value of the average potential increases with a higher proportion of medium-dense region (Supplementary Fig. S8B, -2.8 V) and decreases with a higher proportion of high-dense region (Supplementary Fig. S8C, -1.1 V). In other words, the multiple small pores in the medium-dense region are more effective in storing the printed charges, consistent with the analysis of the porosity effect on the dielectric property in Supplementary Note 9.

**Supplementary Note 11. The pore structure and surface potential for high-permittivity samples.**

This work focuses on enhancing the electro-mechanical properties of the BUE-PVDF materials with a unique charge structure. To further improve and understand this type of material, it is necessary to develop the corresponding characterization method of this BUE structure. However, it is not easy to directly observe the interior charge of the material. Thus, we have found a method to indirectly characterize the charge distribution inside the film by measuring dielectric and surface potential.

As shown in Supplementary Fig. S9A, the pore structure of the high permittivity sample is relatively simple compared to the medium permittivity sample in Supplementary Fig. S8. We observed a significant decrease of about 1.5 V near the pore. The concentration of negative charges around the pore agrees well with the phenomenon observed in Supplementary Fig. S8. This result indicates that the pores can store charges in both medium and high permittivity samples. In other words, the concentration of negative charges near the smaller and medium pores is commonly observed (Supplementary Fig. S8, Supplementary Fig. S9D) in BUE-PVDF samples regardless of their permittivity.

Considering the larger pore size (>10 μm) and pore distance in high permittivity samples, we need to observe the charge distribution with a more extensive range (40×40 μm, Supplementary Supplementary Fig. S9D). However, the surface potential does not decrease in Supplementary Fig. S9E; it increases around these larger pores. This opposite phenomenon is probably related to the depth of the surface pore. When the depth of the pores is deep enough (>2 μm), the positive charge at the interior would transport through the deep pores and neutralize with the negative charge on surface. Thus, the surface potential distribution around the larger pore (Supplementary Fig. S9B) becomes much higher than that around their smaller pore counterpart (Supplementary

Fig. S9A).

To further clarify the character of large pores, we also observed the surface potential distribution in casted samples. As shown in Supplementary Fig. S9C, there are large pores similar to the high permittivity sample in Supplementary Fig. S9B. However, the charges do not tend to accumulate in the vicinity of the pore (Supplementary Fig. S9F), and the surface potential fluctuates only about 100 mV over the entire observation area. Unlike the printed samples, casted samples do not store positive charges during the fabrication process. As a result, the large pores on the surface have difficulty absorbing negative charges (Supplementary Fig. S9D) and lack internal positive charges to increase their surface potential. (Supplementary Fig. S9E).

Generally, we use KPFM to characterize the surface potential and understand BUE-PVDF's internal charge distribution. Since the charge storage capacity in BUE-PVDF originates from the charge accumulation at the pores, the estimation of charge accumulation capacity on different pore types is crucial. With the pore size in Fig. 3D and Supplementary Fig. S8 less than 1-2 μm and pore depth less than 300 nm, the charge accumulation capacity of these small and medium pores can be well characterized by the KPFM method. However, for those high-permittivity samples in Fig. 2C and Supplementary Fig. S7, the size of the pores responsible for the high permittivity (Supplementary Fig. S7I) is about 3-10 μm. Thus, their pore depth is likely to exceed the thickness of the monolayer film (2 μm), resulting in the neutralization of the internal positive charges with the surface negative charges (Supplementary Fig. S9E). At this point, the measured surface potential from KPFM cannot reflect the condition of internal charge density. Therefore, finding an alternative method to estimate the internal charge distribution for high permittivity samples with larger pores is necessary in future studies.

**Supplementary Note 12. Evaluating the effect of pore size on the dielectric property of BUE-PVDF.**

To quantify the effect of pore size and porosity on the improvement of dielectric property in BUE-PVDF, a geometric model is established and exhibited in Supplementary Fig. S10A. For simplification, we assume all pores are uniformly distributed and have a spherical morphology (blue circle) with the same radius, $r$. Due to the free movement of printed charges on the pore surface, we also assume that the printed charges will affect any crystal grain located at a distance less than $D$ from the pore surface (within the range of black dashed circles). For BUE-PVDF

material per unit volume, the total pore volume (i.e., porosity) is:

$$V = n\frac{4}{3}\pi r^3, \tag{S2}$$

where $n$ is the number of pores. The total surface area of pores is:

$$S = n4\pi r^2 . \tag{S3}$$

The effective volume by a single pore is:

$$V_E = \frac{4}{3}\pi\left[\left(r+D\right)^3 - r^3\right]. \tag{S4}$$

Considering that the charge around large pores is not uniformly distributed in KPFM results (Supplementary Fig. S8), larger pores may not store more printed charges. Therefore, assuming that the number of printed charges in each pore is independent of pore size, the accumulated effective volume by all pores is:

$$A_E = nV_E = V\left[\left(1+\frac{1}{r/D}\right)^3 - 1\right]. \tag{S5}$$

Supplementary Fig. S10B shows the effect of pore size on the accumulated effective volume with a constant total pore volume or a constant total surface area. When the total pore volume is stable, the overall dielectric property in BUE-PVDF (estimated by $A_E$) continues to decay rapidly with the increase in pore size. This tendency suggests that the small pores have a much higher stimulation efficiency on crystal grains. Thus, reducing pore size can effectively enhance the dielectric property of BUE-PVDF (Supplementary Fig. S7). Similarly, when the total pore surface area remains constant, the overall dielectric property decays rapidly with small pore size (pore radius is less than twice the affecting distance $D$). However, it remains almost unchanged with a larger pore size.

The pore size data in Supplementary Fig. S8F (with the average pore radius of approximately 4.5 μm and 1.78 μm, respectively) are taken into the above model to assess its reliability. The affecting distance $D$ is solved to be around 3.6 μm, which is close to the radius of the spherulite in Supplementary Fig. S7. This estimated value of $D$ indicates that the trapped space charges within the pore can only affect the adjacent spherulite. Meanwhile, considering:

$$S = 3V / r , \tag{S6}$$

the total pore surface area ratio between these two samples is 2.9/1.78:9.8/4.5 ≈ 0.75. Therefore, a small-pore sample with less total surface area achieves higher permittivity than its counterpart with larger pores.

Although the above model is oversimplified, it provides semi-quantitative guidance for enhancing the dielectric property by controlling porosity. The Eq. (S5) implies that the overall dielectric property is directly proportional to the porosity and approximately inversely proportional to the cube of pore size $r$ in the small size range. The relative permittivity $\varepsilon_r$ for the current BUE-PVDF sample is 7.8 for the sample with an average pore radius of 4.5 μm and a porosity of 9.8%. The relative permittivity $\varepsilon_r$ is enhanced to 12.8 when the average pore radius decreases to 1.78 μm, and the porosity reduces to 2.9%. If the average pore size is further reduced to 1 μm and the porosity is 2%, the relative permittivity $\varepsilon_r$ can be enhanced to 31.8 (like Supplementary Fig. S7D or S7G). The maximum relative permittivity ($\varepsilon_r$=36) in Supplementary Fig. S7G may be reached with an average pore radius of 1 μm and a porosity of about 4%. Therefore, manipulating the porosity configuration of BUE-PVDF to form a more dispersed pore structure throughout the whole film is expected to obtain higher dielectric and electrostrictive properties.

**Supplementary Note 13. The influence of crystallinity on the surface potential and dielectric property.**

Supplementary Fig. S11 summarizes the relationship between crystallinity and surface potential in both groups of samples (H1-H7 by EHD printing, L1-L3 by solvent-casting). As the crystallinity increases from 49.4% to 64.1% for casted samples, the surface potentials are around -350 mV. The crystallinity distribution of H1-H7 is from 56.08% to 67.53%, and the corresponding surface potential ranges from -700 mV to -1100 mV. Generally, the surface potential of the casted sample is significantly higher than that of the printed sample with the same crystallinity. Such a lower surface potential in BUE-PVDF should stem from the accumulation of negative charges on the surface of the BUE structure. As shown in the manuscript Fig. 1A, the interior accumulation of positive charge causes the film's surface to adsorb negative charges, thus decreasing the surface potential.

At the same time, we found that the surface potential of the casted sample hardly changed with the increase of crystallinity. In contrast, when the crystallinity of the printed sample exceeds 62%,

its surface potential decreases significantly with the increase in crystallinity. This tendency shows the different sensitivity of the surface activity on crystallinity between the casted and printed samples.

To clarify the origin of the enhanced permittivity, we also present the relationship between these samples' crystallinity and permittivity in Supplementary Fig. S11B. The permittivity values of both printed and casted samples increase with the crystallinity. However, when the crystallinity of the printed sample is higher than 64%, the slope of the permittivity with the change in crystallinity increases rapidly. Considering that the stored charge of the corresponding sample in Supplementary Fig. S11A substantially increases with crystallinity, we believe that the rapid increase for permittivity after 64% crystallinity in Supplementary Fig. S11B should come from the reduced surface potential. In other words, the charge density of the BUE structure influences the dielectric property more effectively than the crystallinity. Therefore, by varying its stored charge density, BUE-PVDF can achieve a broader range of dielectric property in Fig. 4A than the casted or untreated sample.

**Supplementary Note 14. The comparison with casted PVDF and commercial PVDF.**

We prepared a set of solvent-casted samples and measured their electrostrictive performance using the ferroelectric method. The specific process is as follows. Firstly, a certain weight (0.6507 g) of PVDF powder is prepared, and the corresponding weight (4.3556 g) of solvent NMP is added to obtain a 13 wt.% PVDF ink solution. Next, place the serum bottle containing the solution in an ultrasonic cleaner at 40 °C for 1h, then take it out, and let it stand for a moment to remove air bubbles. Finally, the clear and transparent solution is dropped into a glass mold and spread out naturally. The casted samples were obtained after natural drying at room temperature. The measured relative permittivity is 11.3, $M_{33}$=22.3×10$^{-18}$ m$^2$ V$^{-2}$ (Supplementary Fig. S12A and Fig. S12C). From the SEM image of the casted sample in Supplementary Fig. S12B, the distinct porous structure (porosity up to 23%, pore size generally above 10 μm) should result in the leakage phenomenon represented as the widening of the *D-E* curve in Supplementary Fig. S12A. This porous structure causes a decrease in Young's modulus and an increase in $M_{33}$[5, 18, 19]. The results from Supplementary Fig. S12A to S12C indicate that the solvent-casted method can also achieve high electrostriction due to its unique porous structure. However, the difficulty of curing process control may bring many bubbles, causing the film to become wrinkled and fragile.

Hence, the poor workability and thickness control significantly limit the application prospects of the casted PVDF. In addition to the casted sample, we also measured the electrostrictive coefficient of the commercial sample ($\beta$ phase, polarized, corresponding to the commercial PVDF in Fig. 4C) and found that its $M_{33}$ value was around $8.54\times10^{-18}$ $m^2$ $V^{-2}$ (Supplementary Fig. S12D), which is close to the results reported by Hughes[20] ($10.7\times10^{-18}$ $m^2$ $V^{-2}$). However, this value is still much higher than $0.028\times10^{-18}$ $m^2$ $V^{-2}$ reported by Furukawa *et al.*[21]. This discrepancy may be related to the $\beta$ phase optimization of the sample and the different degrees of their polarization.

**Supplementary Note 15. Estimation of ferroelectric and electrostrictive performances in BUE-PVDF from *S-E* curves**

It should be noted that both the PFM test method in Fig. 4B[5, 22] and the photonic sensing method[8] in Fig. 4C accurately reflect the sample's strain with the varied electric field. However, we could still observe fine differences on the shape of *S-E* curve obtained by each method, especially the linearity around the Y-axis. Their different curve shapes are due to the different polarization within the samples. Theoretically, the electro-deformation $S_{33}$ of an electrostrictive material depends mainly on the applied electric field strength $E$ and the remanent polarization $P_r$[9] as shown below:

$$\begin{aligned} S_{33} &= Q_{33}D^2 = Q_{33}(\varepsilon_r\varepsilon_0 E + P_r)^2 \\ &= 2Q_{33}\varepsilon_r\varepsilon_0 P_r E + Q_{33}P_r^2 + Q_{33}\varepsilon_r^2\varepsilon_0^2 E^2 \end{aligned} \quad \text{(S7)}$$

When the applied electric field increment is *dE*, the corresponding electro-deformation increment $dS_{33}$ is:

$$\begin{aligned} \mathrm{d}S_{33} &= 2Q_{33}\varepsilon_r\varepsilon_0 P_r \mathrm{d}E + 2Q_{33}\varepsilon_r^2\varepsilon_0^2 E \mathrm{d}E \\ &= 2Q_{33}\varepsilon_r\varepsilon_0\left(P_r + \varepsilon_r\varepsilon_0 E\right)\mathrm{d}E \end{aligned} \quad \text{(S8)}$$

In Fig. 4B, we adopted a PFM-based deformation test method. Each data point of the curve ($E_i$, $S_i$) is the peak strain $S_i$ corresponding to a sine wave in the 0-$E_i$ range. The remanent polarization $P_{r,i}$ corresponding to each data point increases with $E_i$. Therefore, according to Eq. (S8), $dS_{33}$ increases approximately linearly with $E$, and the overall $S_{33}$ increases approximately parabolically with $E$. If we select $E_i$ near point 0 in Fig.4B, the corresponding $P_{r,i}$ is also close to 0, so the *S-E* curve is close to a standard electrostrictive parabola.

In the ferroelectric analysis (Fig. 4C), the strain curve is a real-time strain measured by optical methods. In this method, we applied a sinusoidal electric field with a fixed magnitude for cycling loadings. Since the remanent polarization $P_r$ of the sample is determined by the magnitude of the electric field, the $P_r$ at each point in the curve almost remains constant. Considering that the $dS_{33}$ of the BUE-PVDF curve in Fig. 4C decreases with increasing $E$, $P_r$ should be in the opposite direction to $E$. So, when $E \to 0, -P_r >> \varepsilon_r \varepsilon_0 E$. In this case, the slope $dS_{33}/dE$ is approximately constant, that is, the $S_{33}$ increases linearly with $E$ under the condition of low electric field.

**Supplementary Note 16. Previous methods for the improvement on the electrostrictive property in PVDF**

In the past, the electrostrictive property of PVDF has long been considered as an intrinsic property which is mainly susceptible to its carbon chain structure[11, 23].Some of these progresses are still valuable and instructive, which could become good comparisons for our current work. Many research works have significantly enhanced the electrostrictive performance of PVDF by changing their carbon chain structure or degree of cross-linking, such as physical transition (The ice water-quenched 5% P(VDF-HFP) copolymer with $M_{33}$ value 140 ×$10^{-18}$ $M^2$ $V^{-2}$ is reported by Lu *et al.*[24]; the P(VDF-TrFE-CFE) terpolymer modified with bis(2-ethylhexyl) phthalate (DEHP) platisizer with $M_{33}$ value 220 ×$10^{-18}$ $M^2$ $V^{-2}$ is reported by Le *et al.*[25]) and chemical reaction ($M_{33}$ value of 44×$10^{-18}$ $M^2$ $V^{-2}$ achieved in P(VDF-TrFE-CTFE) terpolymer by borane/oxygen initiator is reported by Buckley *et al.*[26]). Unfortunately, these methods impact the original crystal structure, sharply weakening the ferroelectric properties[23]. Moreover, much effort is put into improving electrostrictive performance by inducing relaxor phase in PVDF through electron irradiation (The P(VDF-TrFE) copolymer with $M_{33}$ value 2.3×$10^{-18}$ $M^2$ $V^{-2}$ and relative permittivity value 56.5 by electron irradiation is reported by Zhao *et al.*[27];the P(VDF-TrFE) copolymer with $M_{33}$ value 3.13×$10^{-18}$ $M^2$ $V^{-2}$ and up to 5% longitudinal strain by electron irradiation is reported by Zhang *et al.*[28]) or other surface modification (the P(VDF-TrFE) copolymer with $M_{33}$ value 1.8×$10^{-18}$ $M^2$ $V^{-2}$ by proton irradiation is reported by Guo *et al.*[29]).

**Supplementary Note 17. The linear relation between permittivity and optimized electrostriction in BUE-PVDF**

Supplementary Fig. S13 exhibits the dielectric and electrostrictive properties comparison between the mixed powder samples and other samples in the manuscript. Compared with the

single powder samples (green triangle, Single P.), the range for the $\varepsilon_r$ values of the mixed powder samples (blue circle, S5 series) is extended from 8-18 to 18-35, consistent with the results in Fig. S2. This tendency indicates that the dielectric property of the mixed powder samples can be easily manipulated by adjusting their PVDF concentration. Therefore, we utilize the mixed powder samples in the manuscript (Fig. 2E) to demonstrate the relationship between pore structure and dielectric property with varying ink concentrations.

For the mixed powder samples, each sample's electrostrictive coefficients ($M_{33}$) are generally increased with their permittivity. This increasing slope is close to that of the final optimized samples (red pentagram, S6 series, also shown in Fig. 2H). Still, for other samples, such as S1 series samples with different voltages (light blue pentagons), S3 series samples with different thickness (purple diamonds), and the single powder samples with various concentrations (Single P.), the increasing slope is quite different. It may even reduce to zero or present as a negative value. More importantly, the electrostrictive performances of these samples are much lower than those of the final optimized samples (S6 series) with comparable permittivity. These results prove that the dielectric and electrostrictive properties are unlikely to be simultaneously optimized by controlling only one printing parameter. In other words, optimizing the overall electro-mechanical performances in BUE-PVDF should be governed by multiple printing parameters.

The insufficient optimization of the electrostrictive performance among these samples is probably due to the difference in charge density between the internal and surface regions of the printed film. In the current work, the $M_{33}$ value of the sample is based on the measurement of the film's overall strain. In contrast, the measured relative permittivity of the sample only reflects dielectric property on the surface region (near the detecting electrode)[4, 16]. Therefore, the electrostrictive property can be optimized once the localized charge density at the film's interior is close to its upper limit. This upper limit should be related to the dielectric property on the surface due to the comparable pore structure throughout the whole film. Under such circumstances, the optimized electrostrictive coefficient can be almost increased linearly with the permittivity in Fig. S13 (red shadowed area). Unfortunately, because of multiple factors[30, 31, 32], the printed charge may not be preserved in each layer at the same level during the printing process. Thus, the overall $M_{33}$ values among these samples are usually lower than their theoretical upper limits due to the lower charge density at the film's interior.

The testing results from more samples (black squares in Fig. S13) exhibit a common tendency: the printed sample with similar dielectric properties but prepared with different printing parameters may diverge widely in their electrostrictive performance. Meanwhile, the optimized electrostrictive coefficient (the upper limits of $M_{33}$ in Fig. S13) increases linearly with permittivity. In general, the electrostrictive performance of BUE-PVDF samples still improves with the enhancing permittivity. It is worth noting that the EM properties for most BUE-PVDF samples are much higher than that of the casted or commercial PVDF sample (Fig. S12), demonstrating the effectiveness of BUE structure in improving the EM properties of PVDF.

**Supplementary Note 18. The characteristic of pore structure in the high-permittivity PVDF samples**

In this study, we propose that the electrical-lubricating effect from the stored charge near the pores improves the dielectric property of BUE-PVDF. Thus, the permittivity should be highly correlated with the porosity and pore distribution of the material (Fig. 2E and Fig. S7). Therefore, for the sample with $\varepsilon_r$=56 in Fig. 2H (sample 6-7), its higher permittivity than other samples should originate from the further optimization of the pore structure. As discussed in Note 17, the best optimization of the pore structure in this sample requires comprehensive control of various printing parameters such as printing speed, substrate temperature, air humidity, ink concentration, etc., rather than the variation of single parameter like ink concentration.

To illustrate the importance of pore structure on these high-permittivity samples, we quantify their pore structure parameters from SEM images through a unified measuring method. Fig. S14A and S14B show the mesh-like pattern of the printed samples 5-1 and 5-3 prepared from the mixed-powder ink. There are remarkable differences in localized porosity between the track and the gap regions. Therefore, we must separately measure the porosity $\rho_{track}$, $\rho_{gap}$ and area fraction $Ar_{track}$, $Ar_{gap}$ in the track and the gap regions. Then, the overall porosity $\rho$ of the samples can be expressed as:

$$\rho = Ar_{track}\rho_{track} + Ar_{gap}\rho_{gap} \quad \text{(S9)}$$

$Ar_{track}$ and $Ar_{gap}$ 's evaluation relies on measuring each region's average length from SEM images with low magnification. As shown in Fig. S14A, we mark the boundaries of all track regions based on their distinct porosity and surface height. Then, the average length of each area (illustrated in Fig. S14A) along the X and Y directions, $L_{track,x}$, $L_{gap,x}$, and $L_{track,y}$, $L_{gap,y}$ are

measured by the line intercept method. For each unit cell, the area fraction of each region can be approximately estimated as:

$$\begin{cases} Ar_{gap} = L_{gap,x} \cdot L_{gap,y} / \left[ \left( L_{track,x} + L_{gap,x} \right) \cdot \left( L_{track,y} + L_{gap,y} \right) \right] \\ Ar_{track} = 1 - Ar_{gap} \end{cases} \tag{S10}$$

Similarly, we reproduce samples 6-4 and 6-7 and calculate the area fraction of each region through the corresponding SEM images (Fig. S14C and S14D). These samples are prepared with the same printing parameters as shown in Table S5. The permittivity and electrostrictive coefficient of these reproduced samples (6-4R and 6-7R) are closely comparable to those of the original ones shown in Fig. 2H. It should be noted that we use more than four SEM images for each sample to ensure a reliable measurement of the area fraction.

$\rho_{\mathrm{track}}$ and $\rho_{\mathrm{gap}}$ can be directly measured from the average localized porosity from several high-magnified SEM images. An OpenCV (Open-Source Computer Vision Library) module in the Python library is adopted to identify the porosity in each SEM image. The original image is converted to a binary version with a careful threshold value setting. Hence, the localized porosity of the corresponding image is estimated as the ratio of black pixels (pore areas) to the entire image area. Fig. S14E-S14P show the porosity identification results with tolerable accuracy from the track and the gap region in the samples 5-3, 6-4R, and 6-7R, respectively. Similarly, we selected at least three SEM images for each sample to ensure a reliable estimation of the localized porosity. Suppose there are several different values of localized porosity in the same region type (e.g., track area in Fig. S14B). In that case, we will consider the frequencies of these localized porosity to further amend the overall porosity within the gap/track region.

Supplementary Table S6 summarizes the porosity parameters measured from the mixed powder samples 5-1 and 5-3 and single powder samples 6-4R and 6-7R, respectively. It can be found that except for sample 5-1 with the lowest permittivity value (18), the localized porosity in the track regions of the other three samples (with permittivity value higher than 30) is greater than that in the gap region. Meanwhile, samples with higher localized porosity in the track region tend to have higher overall porosity and more excellent dielectric property. This trend is also reflected in Fig. S7A, D, G, and M. Since the formation of localized pore structure in each region depends on the corresponding curing rates during the printing process, the different curing rates of the track and the gap regions make it unlikely to optimize the pore structures of both regions

simultaneously. Considering $Ar_{\text{track}}$ (the area fraction of the track region) is usually more than 50%, the printing parameters that can optimize the track region's pore structure will be more effective in improving the film's overall porosity and dielectric property.

Note that the above analysis only examines the relationship between pore structure and dielectric property in terms of the average porosity. However, the uniformity of the pore distribution (Fig. 3F), pore size, and pore density (Fig. S10) may also affect the dielectric property. Compared to the mixed powder sample (Fig. S14B), the single powder sample (Fig. S14D) has a uniformly distributed pore structure within its track region (Fig. S7H, S7I, and Fig. S14G). Therefore, sample 6-7R can store more charges through more and smaller pores under similar porosity, thus significantly improve its dielectric performance.

## Supplementary Figures

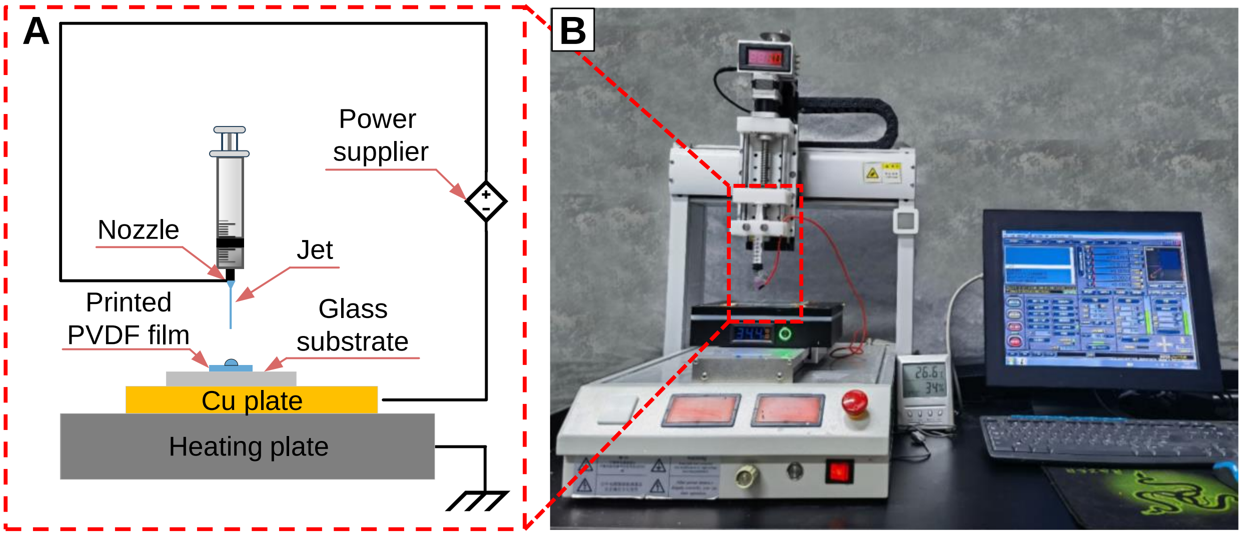


**Supplementary Fig. S1. The set-up of the EHD printing system.** (A) Scheme of the EHD printing system during the printing process. (B) Photograph showing the setup of the substrate and the electrode in the EHD printing system.

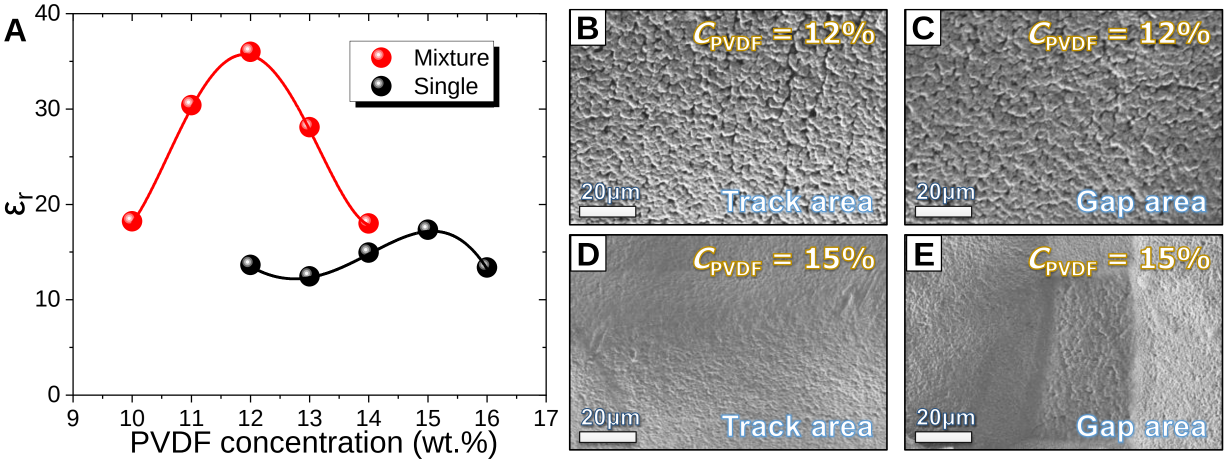


**Supplementary Fig. S2. The comparison of the samples prepared by the single and mixed PVDF powder.** (A) The relationship between permittivity values and the ink concentrations for samples prepared by PVDF ink with single powders and mixed powders over their printable concentration range, respectively. (B) and (C) The pore structure in PVDF sample made by mixed powders at 12 wt.% in its track area and gap area, respectively. (D) and (E) The SEM image exhibiting the pore structure in PVDF sample made by single powder at 15 wt.% in its track area and gap area, respectively.

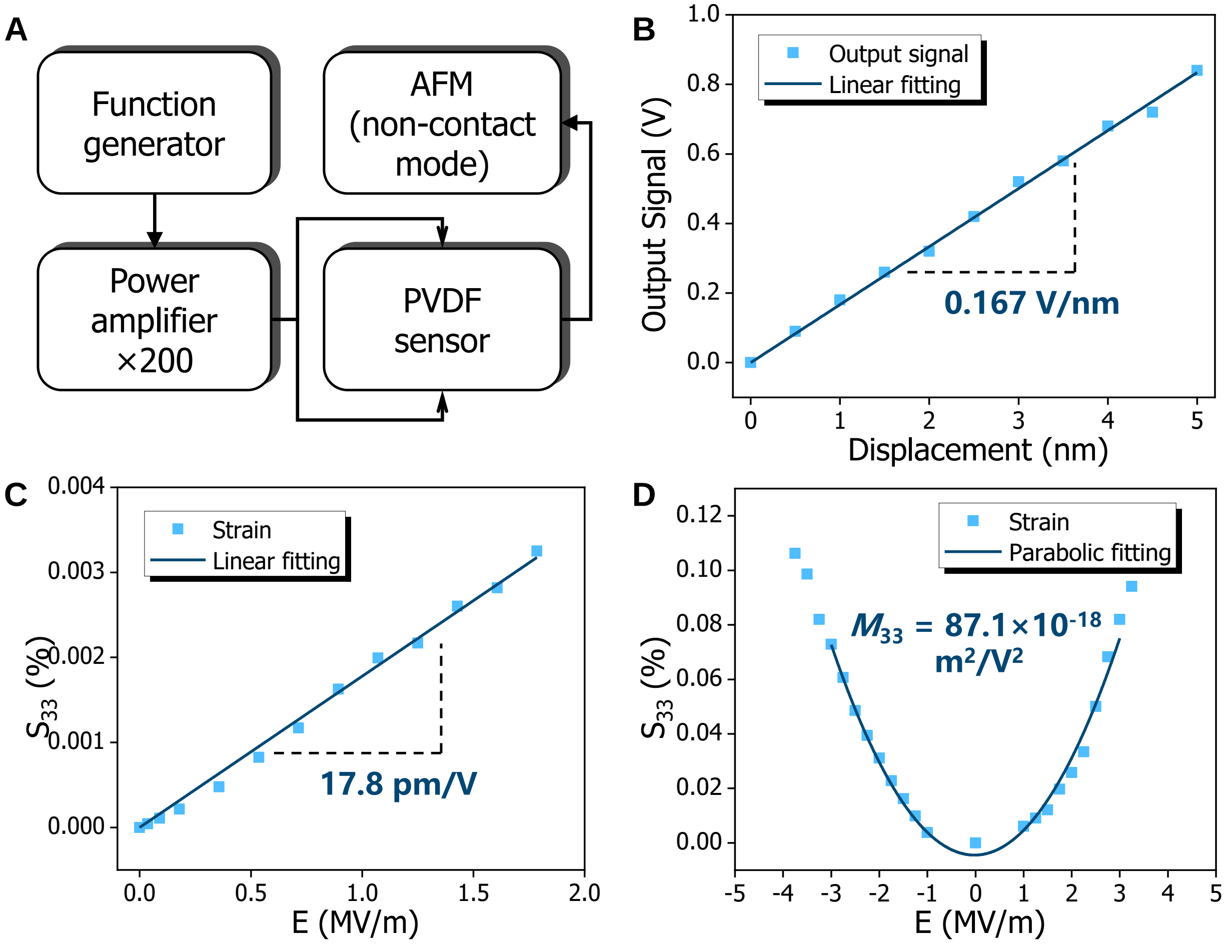


**Supplementary Fig. S3. The calibration of the PFM method and the preliminary exploration of BUE-PVDF.** (A) Schematic diagram of the PFM mode on an AFM platform to detect the small displacement on the surface of PVDF sensor. (B) The calibration curve obtained from the PFM test to show the relationship between the output signal and the displacement of the PZT samples. (C) PFM test result on commercial PVDF sensor. (D) The induced strain under the applied harmonic AC voltage measured by PFM.

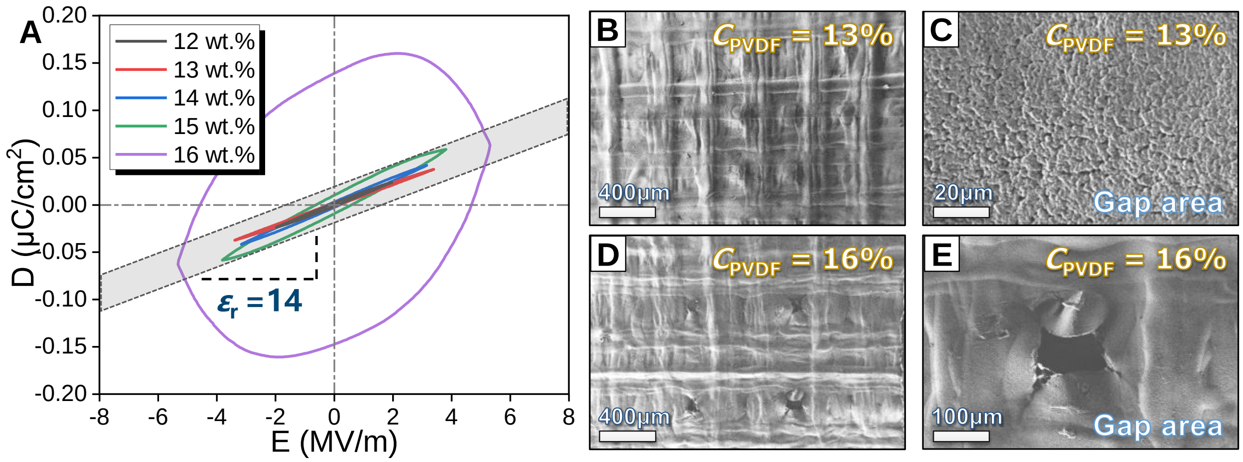


**Supplementary Fig. S4. The charge leakage issue of the printed PVDF.** (A) The *D*-*E* curve and dielectric results of samples with different ink concentrations and conductivities. (B)-(C) The SEM patterns exhibiting the topological and pore structure in a sample at 13 wt.% with different magnifications. (D)-(E) The SEM patterns exhibiting the topological and pore structure in a sample at 16 wt.% with different magnifications.

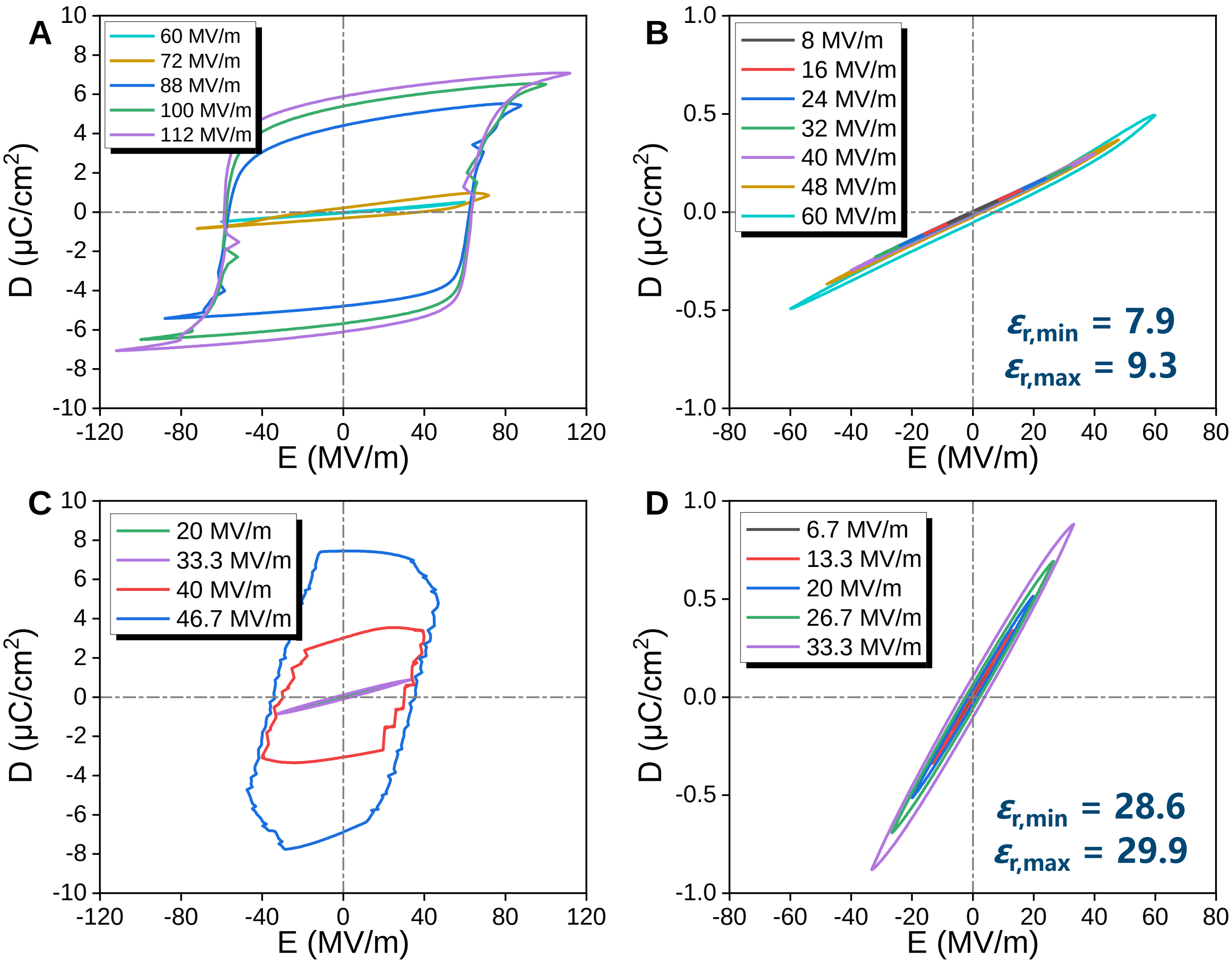


**Supplementary Fig. S5. The comparison of the hysteresis curves between the commercial PVDF and BUE-PVDF.** The polarization hysteresis curves of a commercial $\beta$-phase PVDF film (A) at high electric field and (B) at low electric field, respectively. The polarization hysteresis curves of a BUE-PVDF film (C) at high electric field and (D) at low electric field, respectively.

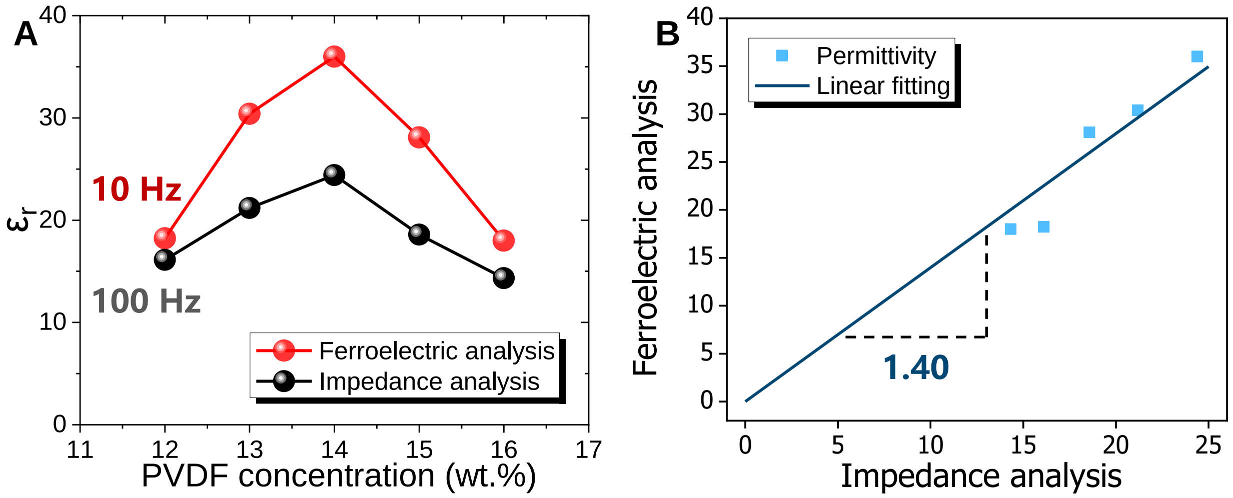


**Supplementary Fig. S6. The comparison of the two methods on their dielectric properties.** (A) Variation of relative permittivity with PVDF concentration by ferroelectric method and impedance method respectively. (B) Comparison of different methods by linear fitting.

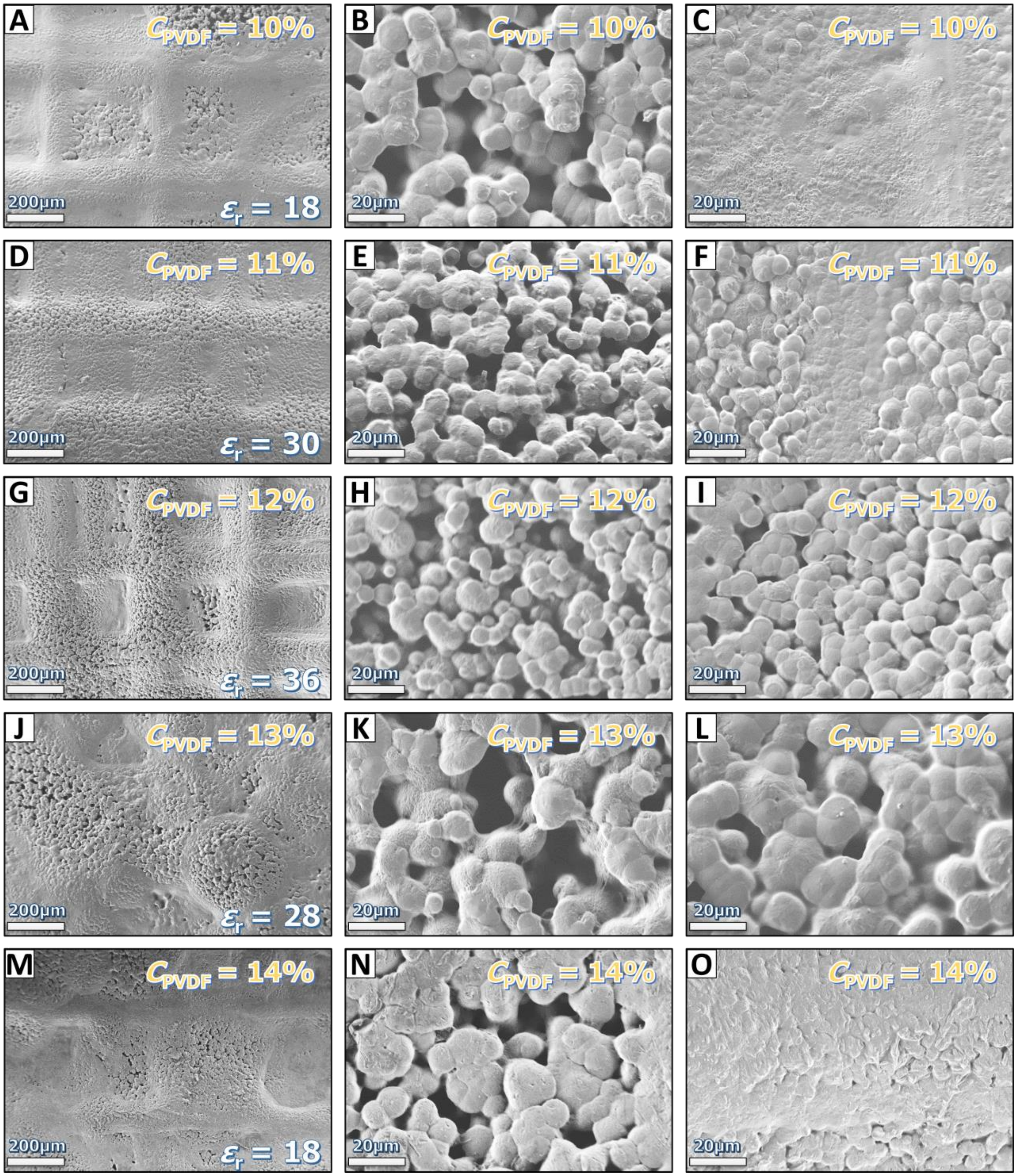


**Supplementary Fig. S7. The SEM images of the printed PVDF samples with varying PVDF concentrations.** The pores distribution with ink track pattern, the corresponding large pores distribution, and the small pores distribution in samples with (A to C) 10 wt.%, (D to F) 11 wt.%, (G to I) 12 wt.%, (J to L) 13 wt.%, and (M to O) 14 wt.% PVDF concentration respectively.

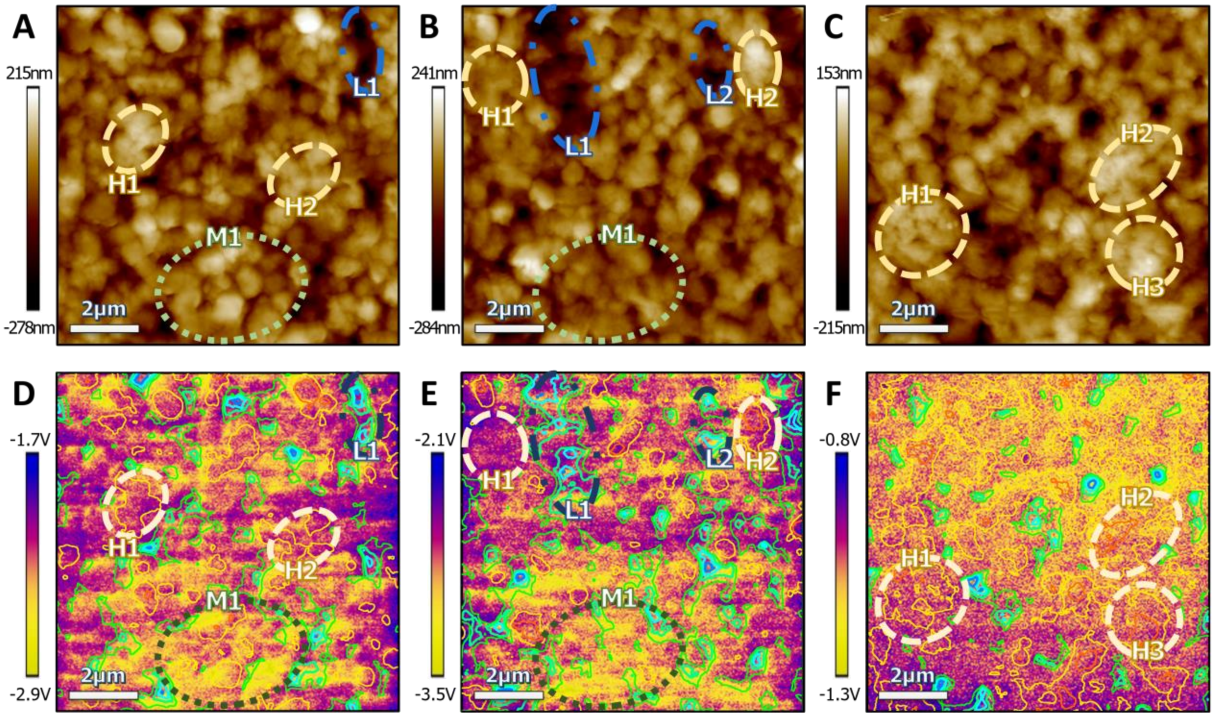


**Supplementary Fig. S8. The comparison diagram of the topography image and potential distribution images for the printed PVDF samples.** The topography image on the surface of BUE-PVDF film and the corresponding potential distribution image for (A to D) sample1, (B to E) sample 2, and (C to F) sample 3 respectively

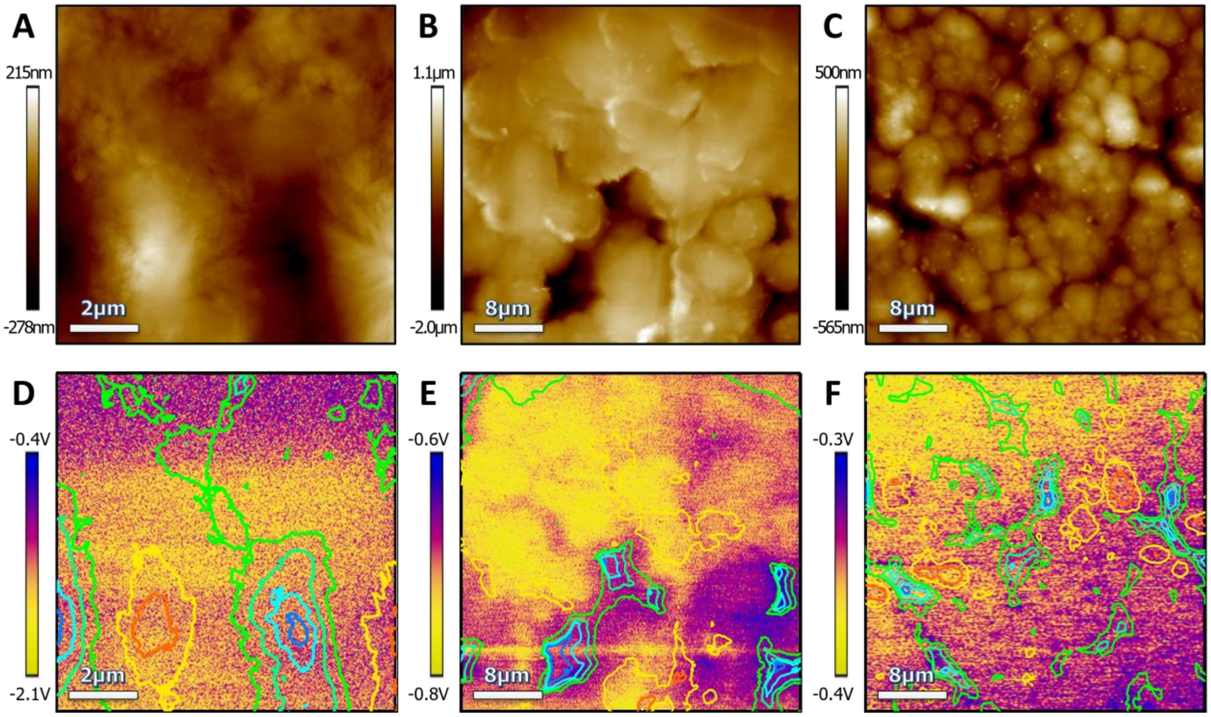


**Supplementary Fig. S9. The comparison diagram of the topography image and potential distribution images for the 12 wt.% PVDF printed high-permittivity sample and casted sample.** The topography image on the surface of BUE-PVDF film and the corresponding potential distribution image for (A and D) in the medium pore region of the printed sample, (B and E) in the large pore region of the printed sample, and (C and F) in the pore region of casted sample, respectively.

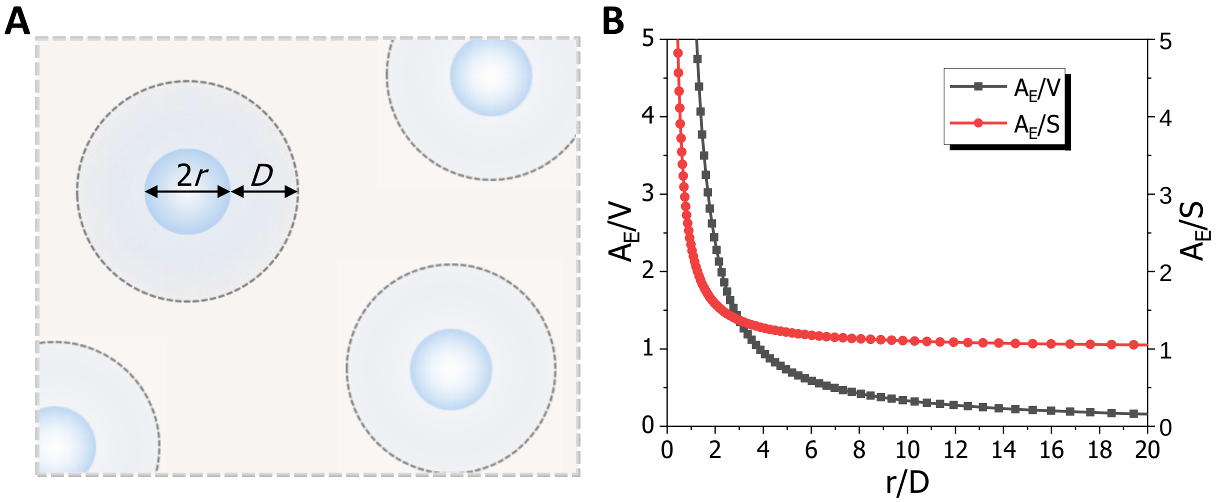


**Supplementary Fig. S10. The schematic of model to evaluate the relaxation effect of the pores with respect to the pore size.** (A) Schematic diagram of pore size and the size of its affecting area. (B) The influence of r/D on the relaxation level of crystal phase per volume and per surface area respectively.

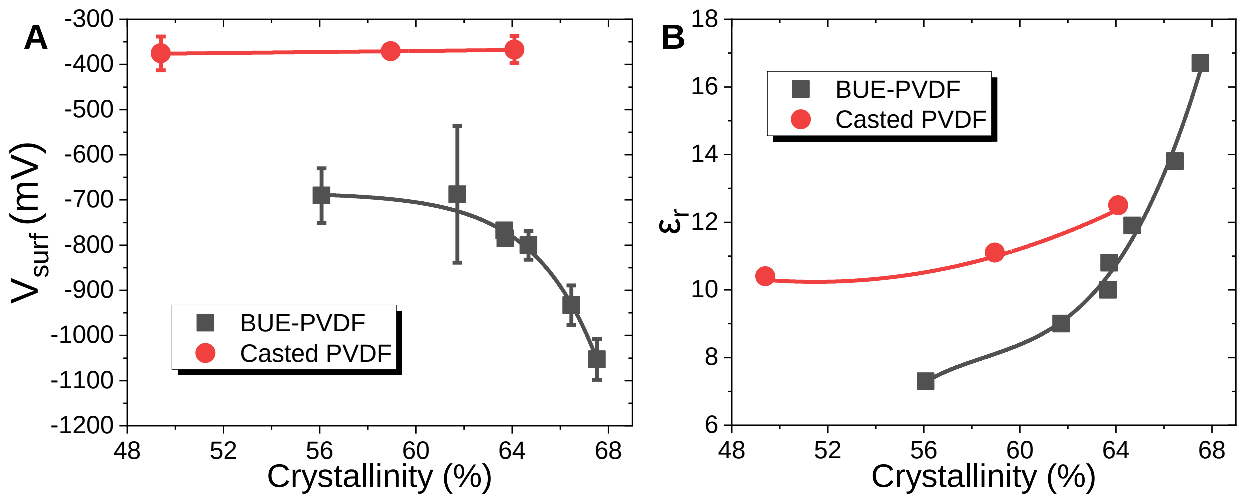


**Supplementary Fig. S11. The influence of crystallinity on the surface potential and dielectric property in the BUE-PVDF and casted PVDF, respectively.** (A) The variation of surface potential of BUE-PVDF (black square) and casted PVDF (red circle) with different crystallinity, respectively. Data are presented as mean ± standard deviation. (B) The variation of relative permittivity of BUE-PVDF (black square) and casted PVDF (red circle) with different crystallinity, respectively.

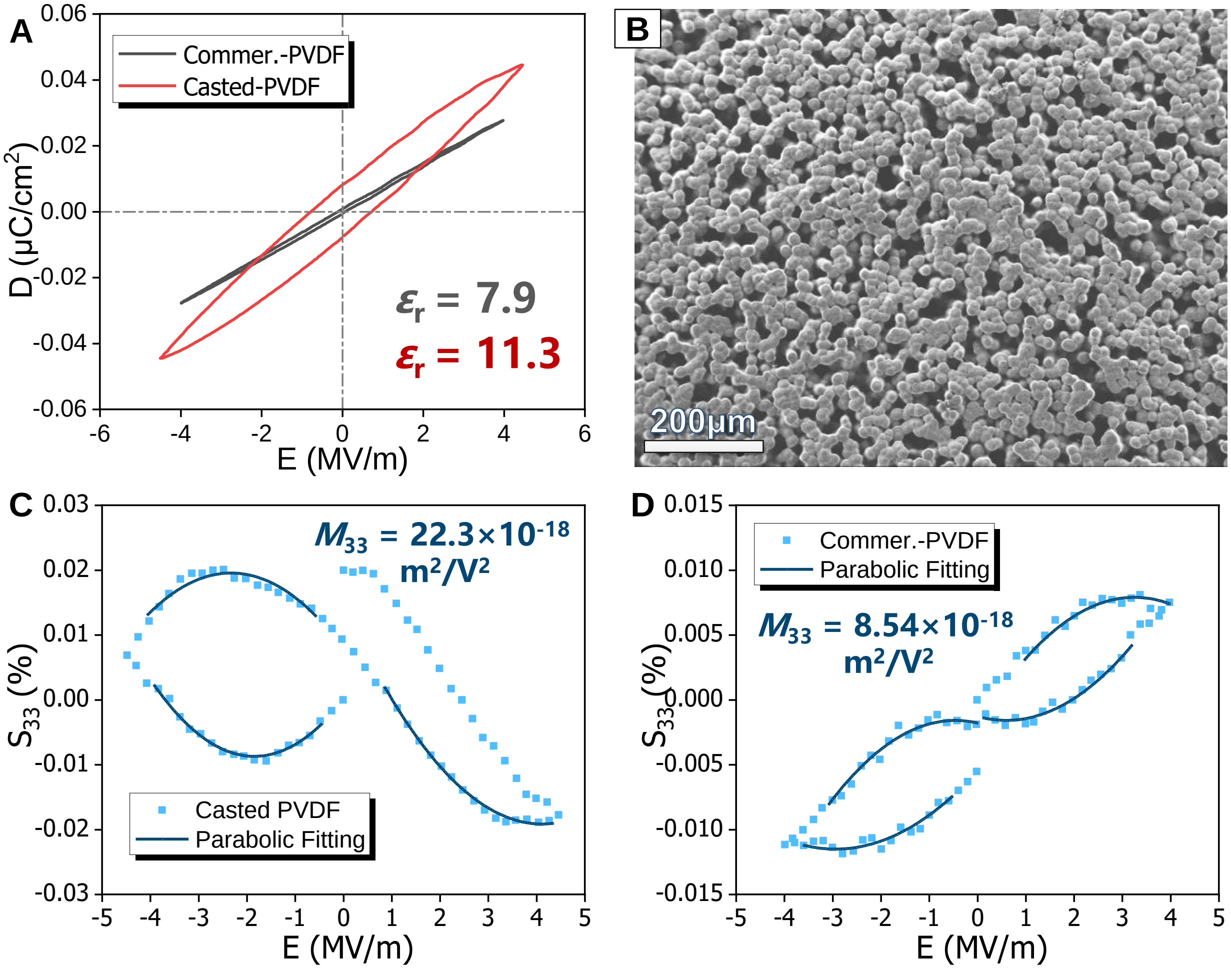


**Supplementary Fig. S12. The comparison of the BUE-PVDF, casted PVDF and commercial PVDF with respect to their dielectric and EM performances.** (A) The polarization hysteresis curves of the casted PVDF samples. (B) The SEM image of the pore structure within the casted PVDF sample. (C) The *S*-*E* curve of the casted PVDF sample. (D) The *S*-*E* curve of the commercial-PVDF sample.

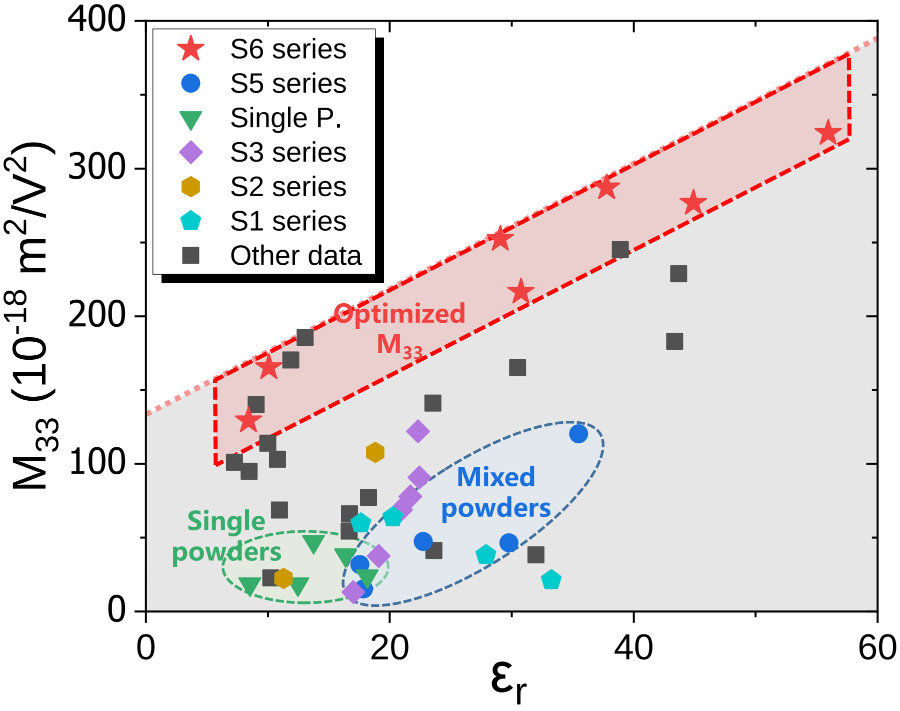


**Supplementary Fig. S13.** The effect of powder blending in sample 5 series on performance improvement in comparison with other sample series.

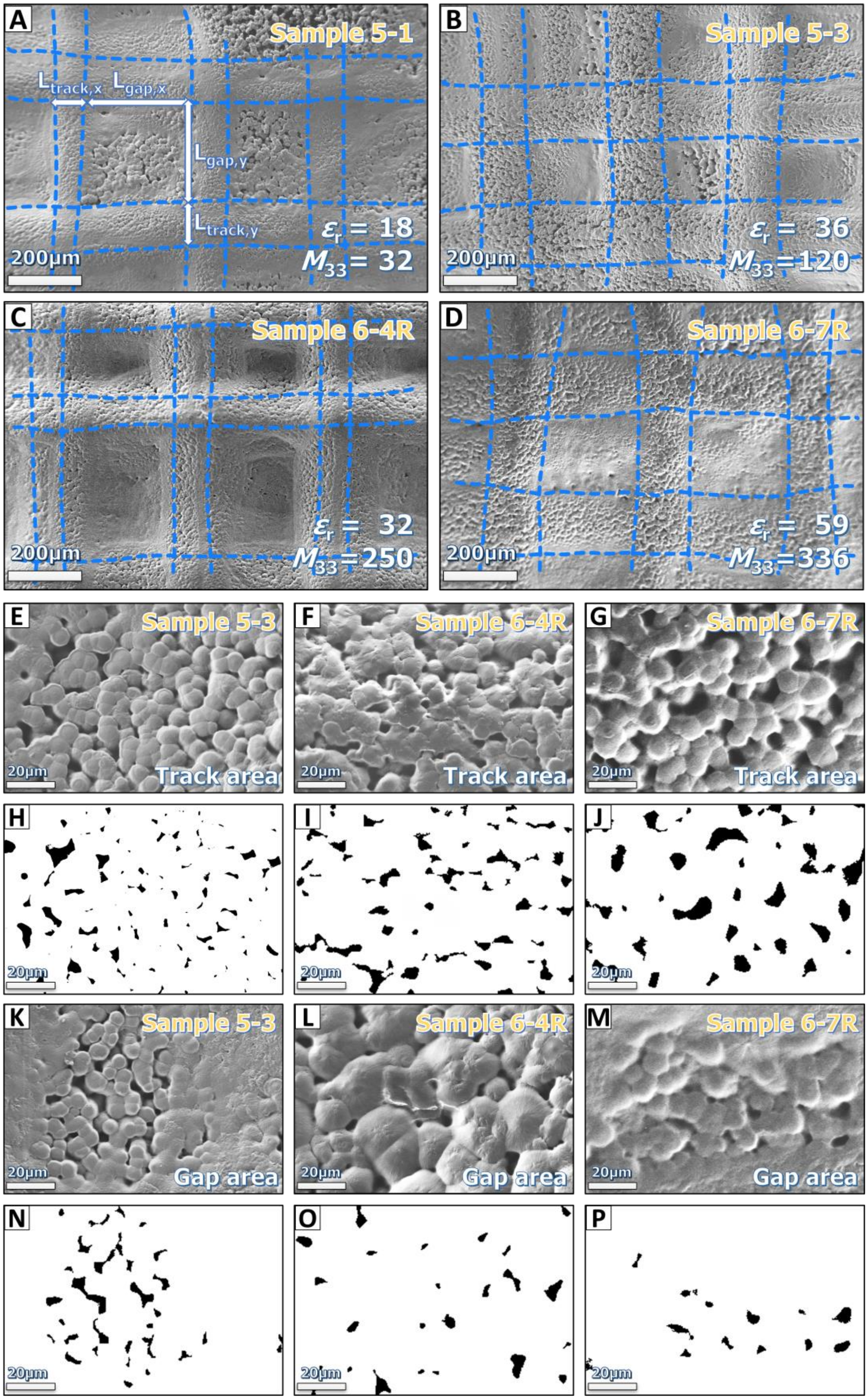
A
Sample 5-1
$L_{track,x}$ $L_{gap,x}$
$L_{gap,y}$
$L_{track,y}$
$\varepsilon_r$ = 18
$M_{33}$ = 32
200μm
B
Sample 5-3
$\varepsilon_r$ = 36
$M_{33}$ = 120
200μm
C
Sample 6-4R
$\varepsilon_r$ = 32
$M_{33}$ = 250
200μm
D
Sample 6-7R
$\varepsilon_r$ = 59
$M_{33}$ = 336
200μm
E
Sample 5-3
20μm
Track area
F
Sample 6-4R
20μm
Track area
G
Sample 6-7R
20μm
Track area
H
20μm
I
20μm
J
20μm
K
Sample 5-3
20μm
Gap area
L
Sample 6-4R
20μm
Gap area
M
Sample 6-7R
20μm
Gap area
N
20μm
O
20μm
P
20μm

**Supplementary Fig. S14. The characteristic and analysis of pore structure in the high-permittivity PVDF samples.** (A-D) The different distribution and size of porosity in the track and the gap regions of the printed PVDF samples corresponding to samples 5-1, 5-3, 6-4R, and 6-7R, respectively. (E-J) The SEM images and their correlated binary images for the samples' track region corresponding to samples 5-3, 6-4R, and 6-7R, respectively. (K-P) The SEM images and their correlated binary images for the sample's gap region corresponding to samples 5-3, 6-4R, and 6-7R, respectively.

## Supplementary Tables

**Supplementary Table S1.** Printing parameters for the PVDF samples correspond to Fig. 1B.

| Sample # | Printing voltage（kV） | Remarks |
|---|---|---|
| 1-1 | - | powder |
| 1-2 | 3 | film |
| 1-3 | 4 | film |
| 1-4 | 5 | film |
| 1-5 | 6 | film |

**Supplementary Table S2.** Printing parameters for the casted and printed samples correspond to Fig. 2B.

| **Sample #** | **Printing voltage (kV)** | **Thickness (µm)** | **Remarks** |
|---|---|---|---|
| 2-1 | - | 39.25 | casted |
| 2-2 | 4 | 42.23 | printed |

**Supplementary Table S3.** Printing parameters for the PVDF samples correspond to Fig. 2C and Fig. 2D.

| Sample # | Concentrations (wt.%) | Printing voltage (kV) | Thickness (μm) | Remarks |
|---|---|---|---|---|
| 3-1 | 13 | 4 | 15 | Higher charge density |
| 3-2 | 13 | 4 | 41 | - |
| 3-3 | 13 | 4 | 53 | - |
| 3-4 | 13 | 4 | 79 | - |
| 3-5 | 13 | 4 | 95 | - |
| 3-6 | 13 | 4 | 113 | - |
| 4-1 | 16 | 4 | 21 | Lower charge density |
| 4-2 | 16 | 4 | 43 | - |
| 4-3 | 16 | 4 | 62 | - |
| 4-4 | 16 | 4 | 85 | - |
| 4-5 | 16 | 4 | 107 | - |
| 4-6 | 16 | 4 | 125 | - |

**Supplementary Table S4.** Printing parameters for the PVDF samples correspond to Fig. 2E.

| Sample # | Concentrations（wt.%） | Relative humidity (%) | Thickness (μm) |
|---|---|---|---|
| 5-1 | 10 | 36 | 43.2 |
| 5-2 | 11 | 31 | 59.2 |
| 5-3 | 12 | 33 | 59.8 |
| 5-4 | 13 | 32 | 45.4 |
| 5-5 | 14 | 35 | 41 |

**Supplementary Table S5.** Printing parameters for the PVDF samples correspond to Fig. 2H.

| **Sample #** | **Relative permittivity** | **Solvent** | **Concentration（wt.%）** | **Substrate temperature (°C)** | **Relative humidity (%)** |
|---|---|---|---|---|---|
| 6-1 | 8.4 | DMF | 10 | 80 | 65 |
| 6-2 | 10.1 | NMP | 14 | 60 | 55 |
| 6-3 | 29.1 | DMF | 10 | 50 | 30 |
| 6-4 | 30.8 | NMP | 13 | 40 | 22 |
| 6-5 | 38.2 | DMF | 10 | 50 | 31 |
| 6-6 | 44.9 | DMF | 11 | 50 | 22 |
| 6-7 | 56.0 | DMF | 10 | 50 | 22 |

**Supplementary Table S6.** The statistical parameters for the pore structures in samples 5-1, 5-3, 6-4R, and 6-7R with different permittivity.

| **Sample #** | $L_{track,x}$ **(μm)** | $L_{track,y}$ **(μm)** | $L_{gap,x}$ **(μm)** | $L_{gap,y}$ **(μm)** | $Ar_{track}$ | $\rho_{track}$ **(%)** | $\rho_{gap}$ **(%)** | $\rho$ **(%)** | $\varepsilon_r$ | $M_{33}$ **($m^2/V^2$)** |
|---|---|---|---|---|---|---|---|---|---|---|
| 5-1 | 117 | 130 | 222 | 225 | 58.5% | 1.5 | 4.0 | 2.5 | 18 | 32 |
| 5-3 | 127 | 132 | 210 | 227 | 60.6% | 7.3 | 4.3 | 6.1 | 36 | 120 |
| 6-4R | 112 | 111 | 280 | 237 | 51.4% | 5.1 | 2.7 | 3.9 | 32 | 250 |
| 6-7R | 127 | 150 | 229 | 216 | 62.1% | 10.2 | 3.7 | 7.7 | 59 | 336 |

## Supplementary Reference


1. Erhard, D. P. et al. Recent advances in the improvement of polymer electret films. *Complex Macromol. Syst. II*, 155-207 (2010).
2. Zheng, J., He, A., Li, J., Han, C. C. Polymorphism control of poly(vinylidene fluoride) through electrospinning. *Macromol. Rapid Commun.* **28,** 2159-2162 (2007).
3. Azzaz, C. M., Mattoso, L. H., Demarquette, N. R., Zednik, R. J. Polyvinylidene fluoride nanofibers obtained by electrospinning and blowspinning: Electrospinning enhances the piezoelectric β-phase–myth or reality? *J. Appl. Polym. Sci.* **138,** 49959 (2021).
4. Jin, L., Li, F., Zhang, S. Decoding the fingerprint of ferroelectric loops: comprehension of the material properties and structures. *J. Am. Ceram. Soc.* **97,** 1-27 (2014).
5. Nawaka, K., Putson, C. Enhanced electric field induced strain in electrostrictive polyurethane composites fibers with polyaniline (emeraldine salt) spider-web network. *Compos. Sci. Technol.* **198,** 108293 (2020)
6. Jesse, S., Baddorf, A. P., Kalinin, S. V. Switching spectroscopy piezoresponse force microscopy of ferroelectric materials. *Appl. Phys. Lett.* **88,** (2006).
7. Lu, H. et al. Statics and dynamics of ferroelectric domains in diisopropylammonium bromide. *Adv. Mater.* **27,** 7832-7838 (2015).
8. Jin, L., Li, F., Zhang, S., Green, D. J. Decoding the fingerprint of ferroelectric loops: comprehension of the material properties and structures. *J. Am. Ceram. Soc.* **97,** 1-27 (2014).
9. Katsouras, I. et al. The negative piezoelectric effect of the ferroelectric polymer poly (vinylidene fluoride). *Nat. Mater.* **15,** 78-84 (2016).
10. Li, X. et al. Polymer electrets and their applications. *J. Appl. Polym. Sci.* **138,** 50406 (2021).
11. Li, F., Jin, L., Xu, Z., Zhang, S. Electrostrictive effect in ferroelectrics: An alternative approach to improve piezoelectricity. *Appl. Phys. Rev.* **1,** 011103 (2014).
12. Kim, B. et al. Electric actuation of nanostructured thermoplastic elastomer gels with ultralarge electrostriction coefficients. *Adv. Funct. Mater.* **21,** 3242-3249 (2011).
13. Zhang, Q. M., Bharti, V., Zhao, X. Giant electrostriction and relaxor ferroelectric behavior in electron-irradiated poly(vinylidene fluoride-trifluoroethylene) copolymer. *Science* **280,** 2101 (1998).
14. Anderson, R. Mechanical stress in a dielectric solid from a uniform electric field. *Phys. Rev. B* **33,** 1302 (1986)..
15. Furukawa, T., Nakajima, K., Koizumi, T., Date, M. Measurements of nonlinear dielectricity in ferroelectric polymers. *Jpn. J. Appl. Phys.* **26,** 1039 (1987).
16. Mellinger, A. Dielectric resonance spectroscopy: a versatile tool in the quest for better piezoelectric polymers. *IEEE Trans. Dielectr. Electr. Insul.* **10,** 842 (2003).
17. Wentink Jr, T. Properties of polyvinylidene fluoride. I. dielectric measurements. *J. Appl. Phys.* **32,** 1063-1064 (1961).
18. Assagra, Y., Altafim, R., Carmo, J., Altafim, R., Gerhard, R. A new route to piezo-polymer transducers: 3D printing of polypropylene ferroelectrets. *IEEE Trans. Dielectr. Electr. Insul.* **27,** 1668 (2020).

19. Liu, Q., Richard, C., Capsal, J. F. Control of crystal morphology and its effect on electromechanical performances of electrostrictive P(VDF-TrFE-CTFE) terpolymer. *Eur. Polym. J.* **91,** 46-60 (2017).
20. Hughes, O. R. Frequency dependence of hysteresis associated with the electromechanical performance of PVDF film. *J. Polym. Sci. Part B: Polym. Phys.* **45,** 3207-3214 (2007).
21. Furukawa, T., Seo, N. Electrostriction as the origin of piezoelectricity in ferroelectric polymers. *J. Appl. Phys.* **29,** 675 (1990).
22. Bonnell, D. A., Kalinin, S. V., Kholkin, A., Gruverman, A. Piezoresponse force microscopy: a window into electromechanical behavior at the nanoscale. *MRS Bull.* **34,** 648-657 (2009).
23. Kochervinskii, V. V. New electrostriction materials based on organic polymers: A review. *Crystallogr. Rep.* **54,** 1146 (2009).
24. Lu, X., Schirokauer, A. Giant electrostrictive response in poly(vinylidene fluoride-hexafluoropropylene) copolymers. *IEEE Trans. Ultrason. Ferroelectr. Freq. Control* **47,** 1291-1295 (2000).
25. Le, M. Q. et al. All-organic electrostrictive polymer composites with low driving electrical voltages for micro-fluidic pump applications. *Sci. Rep.* **5,** 11814 (2015).
26. Buckley, G., Roland, C., Casalini, R., Petchsuk, A., Chung, T. Electrostrictive properties of poly(vinylidenefluoride-trifluoroethylene-chlorotrifluoroethylene). *Chem. Mater.* **14,** 2590-2593 (2002).
27. Zhao, X.-Z. et al. Electromechanical properties of electrostrictive poly(vinylidene fluoride-trifluoroethylene) copolymer. *Appl. Phys. Lett.* **73,** 2054-2056 (1998).
28. Zhang, Q., Cheng, Z. Y., Bharti, V., Xu, T. B., Gross, S. J. Piezoelectric and electrostrictive polymeric actuator materials. *Proc. SPIE on Smart Struc. Mater.* **3987,** 34-50 (2000).
29. Guo, S., Zhao, X.-Z., Zhou, Q., Chan, H. L. W., Choy, C. L. High electrostriction and relaxor ferroelectric behavior in proton-irradiated poly(vinylidene fluoride-trifluoroethylene) copolymer. *Appl. Phys. Lett.* **84,** 3349-3351 (2004).
30. Mellinger A. Charge storage in electret polymers: mechanisms, characterization and applications. (Universität of Potsdam, Potsdam, 2004).
31. Belhora F, Guyomar D, Mazroui MH, Hajjaji A, Boughaleb Y. Thickness effects of electret and polymer for energy harvesting: Case of CYTOP- CTLM and polyurethane. *Eur. Phys. J. Plus* **130**, 1-9 (2015).
32. Collins G, Federici J, Imura Y, Catalani LH. Charge generation, charge transport, and residual charge in the electrospinning of polymers: A review of issues and complications. *J. Appl. Phys.* **111,** 044701 (2012).
