# Supplementary material for: Ultra-high electrostriction and ferroelectricity in poly (vinylidene fluoride) by ‘printing of charge’ throughout the film
Source: Nat Commun. 2025 Jan 16;16:744. doi: 10.1038/s41467-025-56064-w (PMC11739654; doi:10.1038/s41467-025-56064-w)
Supplement: Supplementary file 2 — Description of Additional Supplementary Files [file 41467_2025_56064_MOESM2_ESM.pdf]

## Description of Additional Supplementary Files

**Supplementary Movie 1. The distribution of pores at the interior of BUE-PVDF film from Micro-CT observation.** The cross-sections of the reconstructed region (200 μm × 200 μm × 28 μm) along the thickness direction of the sample with 9.8 % porosity in Fig. 3F. Note that the 272 μm × 206 μm label on the left side marks the size of the video, rather than the size of the reconstructed region.
